# Novel Sequence Feature of SecA Translocase Protein Unique to the Thermophilic Bacteria: Bioinformatics Analyses to Investigate Their Potential Roles

**DOI:** 10.3390/microorganisms8010059

**Published:** 2019-12-29

**Authors:** Bijendra Khadka, Dhillon Persaud, Radhey S. Gupta

**Affiliations:** Department of Biochemistry and Biomedical Sciences, McMaster University, Hamilton, ON L8N 3Z5, Canada; khadkab@mcmaster.ca (B.K.); persaudk@mcmaster.ca (D.P.)

**Keywords:** novel sequence features of SecA from thermophilic bacteria, phylogenetic analysis, conserved signature indels, molecular dynamics simulations of *Thermotoga maritima* SecA, conserved water molecules

## Abstract

SecA is an evolutionarily conserved protein that plays an indispensable role in the secretion of proteins across the bacterial cell membrane. Comparative analyses of SecA homologs have identified two large conserved signature inserts (CSIs) that are unique characteristics of thermophilic bacteria. A 50 aa conserved insert in SecA is exclusively present in the SecA homologs from the orders *Thermotogales* and *Aquificales*, while a 76 aa insert in SecA is specific for the order *Thermales* and *Hydrogenibacillus schlegelii*. Phylogenetic analyses on SecA sequences show that the shared presence of these CSIs in unrelated groups of thermophiles is not due to lateral gene transfers, but instead these large CSIs have likely originated independently in these lineages due to their advantageous function. Both of these CSIs are located in SecA protein in a surface exposed region within the ATPase domain. To gain insights into the functional significance of the 50 aa CSI in SecA, molecular dynamics (MD) simulations were performed at two different temperatures using ADP-bound SecA from *Thermotoga maritima*. These analyses have identified a conserved network of water molecules near the 50 aa insert in which the Glu185 residue from the CSI is found to play a key role towards stabilizing these interactions. The results provide evidence for the possible role of the 50 aa CSI in stabilizing the binding interaction of ADP/ATP, which is required for SecA function. Additionally, the surface-exposed CSIs in SecA, due to their potential to make novel protein-protein interactions, could also contribute to the thermostability of SecA from thermophilic bacteria.

## 1. Introduction

Thermophilic organisms (bacteria) are of great scientific interest due to their ability to grow at temperatures well above 60 °C [1,2,3,4,5]. The thermostability of the proteins from model organisms such as *Thermotoga maritima, *which can survive within a wide temperature range of 55–90 °C, has been an area of intense research interest [5,6,7]. Thermostability of the protein has practical applications in industrial settings, biotechnologies, and bio-refining [1,2,3,4,5,8]. Specifically, within industrial settings, the higher temperature stability of these protein catalysts allows for reactions at higher temperatures resulting in decreased contamination concerns and overall faster reaction speeds [9]. An example in this regard includes widespread use of enzyme *Thermus aquaticus *(*Taq*) polymerase in the technique of polymerase chain reaction (PCR) [10]. Several comparative studies have shown that the thermostability of the proteins from thermophilic groups of organisms can be attributed to various characteristics [7,11,12]. One prevalent characteristic is the increase in the presence of ion-pair interactions in thermophilic organisms [11,13]. The increase in ion-pair interactions is due to a higher composition of charged amino acids such as lysine (Lys), arginine (Arg), glutamic acid (Glu) and asparagine (Asn) in the proteins from thermophilic bacteria when compared to those from mesophilic bacteria [11,13]. Sequence and structural characteristics such as presence of insertions and deletions, proline substitutions, closer packing of water-accessible surface residues, and increase in helical contents and hydrogen bonds has also been suggested to contribute towards increase in the thermostability of thermophilic proteins [14,15,16]. Evidently, the characteristics providing thermostability to proteins can exist in various forms, and further understanding protein features and characteristics that likely contribute towards the thermostability of proteins is of much interest [13,17].

Within the domain Bacteria, hyperthermophilic organisms are mainly present in three bacterial phyla viz. *Aquificae*, *Deinococcus*–*Thermus*, and *Thermotogae* [4,9,18,19,20,21]. The members from these phyla, which contain some of the most hyperthermophilic organisms known (e.g., *Thermotoga maritima, Thermus aquaticus,* and *Aquifex aeolicus*), are notable for their thermostable enzymes [1,2,5,9,22]. However, of these three phyla, while the phylum *Aquificae* is primarily comprised of thermophilic–hyperthermophilic organisms [18,20], in the other two phyla, hyperthermophilicity is a shared characteristics of species from only some orders (viz. *Thermotogales* and *Thermales*) of these bacteria [21,23,24,25,26]. Our earlier comparative analyses on protein sequences from members of these three phyla have identified large numbers of molecular markers in the form of conserved signature indels (CSIs) in different proteins which are specifically shared characteristics of different members from each of these three phyla (viz. *Thermotogae*, *Aquificae,* and *Deinococcus*–*Thermus*) of bacteria and their suborders [4,20,23,27,28,29,30]. Although the CSIs that we have previously identified provide very useful means for distinguishing members of these phyla from each other, as well as other groups of bacteria, it is unclear if any of these genetic/biochemical changes play any role in the thermostability of these organisms. However, in the present study we describe the identification and analysis of two large CSIs in the homologs of SecA proteins, which due to their unique shared presence in members from two different phyla of hyperthermophilic bacteria are indicated to play an important role in the thermostability of this protein.

SecA is a conserved ATPase whose homologs are well-preserved among all bacteria [31,32]. It constitutes a major molecular motor for the ATP-driven secretion of pre-proteins across the bacterial membrane in conjunction with the protein translocation complex SecYEG [33,34]. Previous biochemical and structural studies on SecA from thermophilic bacteria have contributed significantly towards understanding the overall architecture and function of the protein [35,36,37]. However, it remains unclear whether the presence of any unique sequence feature(s) in SecA contributes to its stability or for the functioning of SecA translocase at high temperatures. In the present study, we describe the identification and analysis of two large CSIs in the SecA proteins which are uniquely found in the SecA homologs from two different phyla of hyperthermophilic bacteria. One of these CSIs, consisting of an insert of 50 aa in a conserved region, is specifically found in the SecA homologs from all members of the order *Thermotogales* (phylum *Thermotogae*) and *Aquificales* (phylum *Aquificiae*), while another large 76 aa insert has uniquely shared characteristics of the SecA homologs from the order *Thermales *(phylum *Deinococcus*–*Thermus*) and in *Hydrogenibacillus schlegelii*, a thermophilic bacterium from the phylum *Firmicutes*. The shared presence of these large and unique sequence features in the SecA proteins from only the hyperthermophilic members of these phyla strongly suggests that the identified genetic/biochemical changes are related to the thermophilic characteristics of these organisms and they should be playing an important role in the thermostability of this protein.

We report here the results of phylogenetic studies examining the evolution of these large CSIs in the thermophilic organisms, as well as the sequence compositions, structural features, and location of these CSIs in the SecA protein structure using the homology models and available structural information. Lastly, to gain some insights into the functional significance of the 50 aa CSI in SecA protein (which is uniquely found in members of the orders *Thermotogales *and *Aquificales*), we have performed molecular dynamics (MD) simulation at two different temperatures (303.15 K and 363.15 K) using the available SecA structure (*Tm*SecA) from *T. maritima,* which contains this large CSI. The results from MD simulation studies identify a conserved network of water molecules whose interaction with the bound nucleotide in the SecA protein is mediated/stabilized by certain conserved residues from this CSI in *Tm*SecA. The significance of these observations in the context of the specificity of the identified CSIs for thermophilic organisms are discussed.

## 2. Materials and Methods

### 2.1. Identification of Conserved Signature Indels (Insertions/Deletions) and Phylogenetic Analysis

The described CSIs in the SecA proteins were identified as described in earlier work [28,38,39]. In brief, BLASTp searches were carried out on the SecA protein sequences from Thermotogae and other thermophilic organisms. Based on these BLASTp searches, sequences of SecA proteins were retrieved from 10–15 organisms representing different thermophilic phyla as well as a similar number of sequences from members of other bacterial phyla. Multiple sequence alignments (MSAs) of the retrieved sequences were created using ClustalX 2.1 [40,41]. These sequence alignments were examined for the presence of conserved inserts or deletions (i.e., indels), which were specifically found in thermophilic organisms, and which were flanked on both sides by at least 5 conserved residues in the neighboring 30–40 aa. More detailed BLASTp searches on the sequence regions containing the indels of interest and their flanking 40–50 aa were then conducted against the NCBI non-redundant (nr) database to determine the specificity of the identified indels. For the indels of interest, the signature files shown here were created using SIG_CREATE and SIG_STYLE programs (from www.GLEANS.net). Unless otherwise indicated, all of the reported CSIs are specific for the group of interest, and similar CSIs were not observed in homologs from any other bacterial species within the top 500 BLASTp hits examined.

For phylogenetic studies, protein sequences for SecA homologs for species from different groups of interest were obtained by means of BLASTp searches. A multiple sequence alignment of these protein sequences was constructed using ClustalX 2.1 [40]. Poorly aligned regions from the sequence alignment were removed using Gblocks 0.91b program [42], leaving 518 aligned amino acids (without any sequence gaps) for phylogenetic analysis. A maximum-likelihood phylogenetic tree based on 500 bootstrap replicates of this sequence alignment was constructed using MEGA 6 [43] based on the Jones–Taylor–Thornton (JTT) model [44].

### 2.2. Homology Modelling of SecA Homologs and Structural Analysis of CSIs

The homology models of the *Thermotoga maritima* and *Thermus thermophilus* SecA proteins lacking the 50 aa CSI were created using an in-house pipeline, “GlabModeller,” as described in earlier work [45,46,47,48]. The available crystal structures of SecA from *T. maritima* (PDB ID: 4YS0) and *T. thermophilus* (PDB ID: 2IPC) were used as templates [35,36]. In brief, for homology modeling, the sequence alignments between target and template proteins were carried out using the align2D module from the MODELLER, which is integrated and streamlined in the GlabModeller tool. The resulting alignments were carefully analyzed and modified manually to ensure the reliability of the location of CSIs. For each target protein, 500 models were ranked on the basis of their Discrete Optimized Protein Energy (DOPE) scores [49]. Selected models were then refined using ModRefiner [50]. The stereo-chemical properties of the final models were assessed using three independent servers which include RAMPAGE [51], ERRAT [52], PROSA [53,54], and VERIFY3D [55]. These applications utilize a dataset of refined structures to evaluate the statistical significance of the model conformation, location, and environment of each amino acid sequence and overall structural stability. The resultant models were then used to explore the structural changes associated with the CSI. The superimposition of the validated models with the template structures was carried out using PyMOL (Version 1.7.4; Schrödinger, LLC.) to examine the structure and location of identified CSIs in the SecA structures.

### 2.3. Molecular Dynamics Simulations

The all-atom molecular dynamics simulations were performed using (Groningen machine for chemical simulations) GROMACS 5.1.2 software [56,57] with the all-atom CHARMM36 force field for SecA, ADP, ions together with 3-points (TIP3P) water model and added ionic strength to mimic the physiological environment [58]. The atomic coordinates of the *T. maritima *SecA (PDB ID: 4YS0) resolved at 1.85 Å were obtained from the protein data bank (PDB) and the homology model of *Tm*SecA lacking CSI was utilized. The potential energy of the system was minimized using a 50,000-step steepest descendent method to relax the system and to avoid any steric clashes. Missing amino acid residues in the crystal structure of *Tm*SecA were identified and fixed using the MODELLER implemented in the GlabModeller tool. After energy minimization, the system was equilibrated with isothermal–isochoric/NVT (constant number of particles, volume, and temperature) ensemble and then 100 ns of MD simulation in the isothermal–isobaric/NPT (constant number of particles, pressure, and temperature) ensemble using the Nose–Hoover thermostat and Parrinello–Rahman barostat [59,60,61]. Comparative analysis of the difference in binding affinity of ADP towards *T. maritima* SecA with CSI and without CSI was carried out. The *T. maritima *SecA with ADP was simulated under two different reference temperatures of 303.15 K and 363.15 K. These large temperature gaps were selected to investigate the effect of temperature on the *Tm*SecA dynamics during the simulation period. The root mean square deviation (RMSD) and hydrogen bond interaction calculations were carried out using the GROMACS utilities. All MD simulation runs were carried out using our local GROMACS certified graphical processing unit (GPU) accelerated high-performance computing system obtained from EXXACT Corporation [62]. A total of 1000 snapshots were extracted for every 100 ps from the 100 ns MD trajectories to analyze the dynamics of water molecules near the CSI-containing region. The analyses of the structures obtained from trajectories were carried out using the various utilities of the GROMACS [56], VMD [63], and PyMOL (www.pymol.com).

## 3. Results

### 3.1. Identification of Conserved Signature Indels in SecA Homologs from Thermotogales, Aquificales, and Thermales and their Phylogenetic Implications

**Figure 1:**
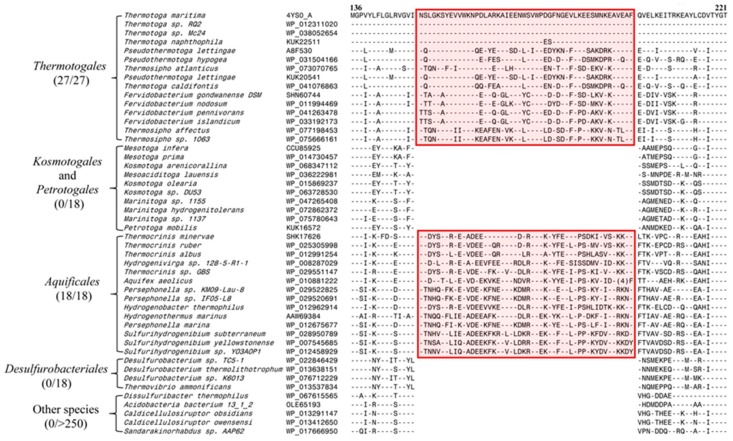
Excerpts from the sequence alignment of SecA protein showing a 50 aa conserved insert that is a distinctive characteristic of species from the orders *Thermotogales* and *Aquificales* but absent in the homologs from all other bacteria. The numbers in the parenthesis below the orders’ names indicate the number of species from these orders for which SecA homologs were identified and how many of them contained the indicated insert. The dashes (-) in the sequence alignment denote sequence identity with the amino acid shown on the top line. The accession numbers of the protein sequences are provided in the second column and numbers on top of the sequence alignment indicate the position of this sequence in *T. maritima*.

Our comparative analysis of SecA protein sequences from different bacterial groups has identified several CSIs in SecA protein that are specific for particular groups/taxa of bacteria. However, the present study focuses on our identification of two large CSIs in the SecA protein, which are uniquely found in different homologs from the thermophilic–hyperthermophilic phyla of bacteria. The first of these CSIs shown in Figure 1 is a 50 aa insertion in SecA homologs that is uniquely shared by members of the order *Thermotogales *and *Aquificales*. As seen from Figure 1, within the phylum *Thermotogae*, this CSI is a shared characteristic of all members from the order *Thermotogales*, but it is not found in any of the species from the orders *Peterotogales* and *Kosmotogales*. It should be noted in this regard, that within the phylum *Thermotogae*, the order *Thermotogales* encompasses all of the thermophilic–hyperthermophilic organisms, whereas the other two orders lacking this CSI are comprised of mesophilic organisms [4,23,26,64]. Thus, within the phylum *Thermotogae*, this CSI is uniquely found in the organisms which are thermophilic–hyperthermophilic [27,28]. Interestingly, in addition to the *Thermotogales*, this large CSI is also commonly shared by different species belonging to the order *Aquificales* from the phylum *Aquificae*, which is also comprised exclusively of hyperthermophilic organisms. However, within this phylum members belonging to the order *Desulfurobacteriales*, which are strict anaerobes [20,65], do not contain this large insert.

**Figure 2:**
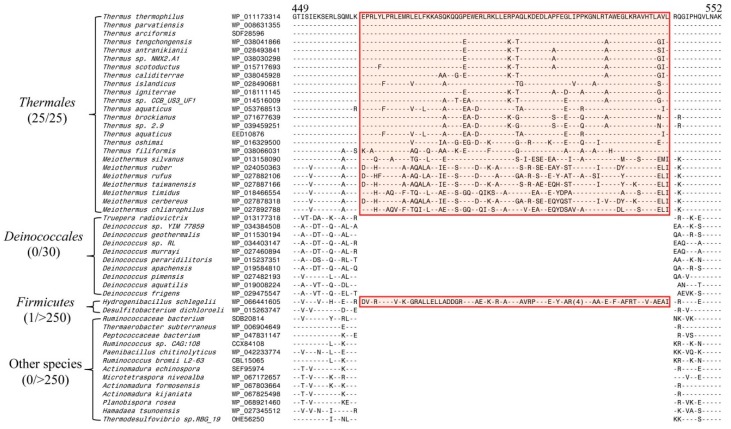
Partial sequence alignment of SecA showing a 76 aa conserved insert that is a unique characteristic of species from the order *Thermales* and *H. schlegelii* but absent from the SecA homologs from all other bacteria. Except for *H. schlegelii*, no other *Firmicutes* species contained this insert. Other information concerning this sequence alignment is the same as in Figure 1.

The second large CSI in SecA protein that we have identified is a 76 aa insertion in a conserved region (see Figure 2), which is commonly shared by all SecA homologs from members of the order *Thermales* belonging to the phylum *Deinococcus*–*Thermus*. The phylum *Deinococcus*–*Thermus* contains two extensively studied orders of extremophilic microorganisms i.e., *Deinococcales* and *Thermales* [21,30]. Of these two orders, the order *Thermales* is comprised exclusively of organisms that are thermophilic and hyperthermophilic [21,30], whereas members of the order *Deinococcales* are known for their high degree of radiation resistance [24,30,66]. Interestingly, this large insert in SecA is found only in different members of the order *Thermales* but not in any of the homologs from *Deinococcales*. Further, in addition to the members from the order *Thermales, *this insert in SecA is also commonly shared by the species *Hydrogenibacillus schlegelii, *which is a thermophilic bacterium belonging to the phylum *Firmicutes* [67,68,69]. Thus, both these large CSIs in SecA are only found in members of different main orders/phyla of bacteria that contain thermophilic-hyperthermophilic organisms. Except for the thermophilic-hyperthermophilic organisms, these inserts are not present in any other SecA homologs. Thus, the indicated CSIs were further examined in the context of their role in thermostability.

### 3.2. Phylogenetic Analysis of the SecA Proteins to Investigate the SHARED Presence of CSIs

**Figure 3:**
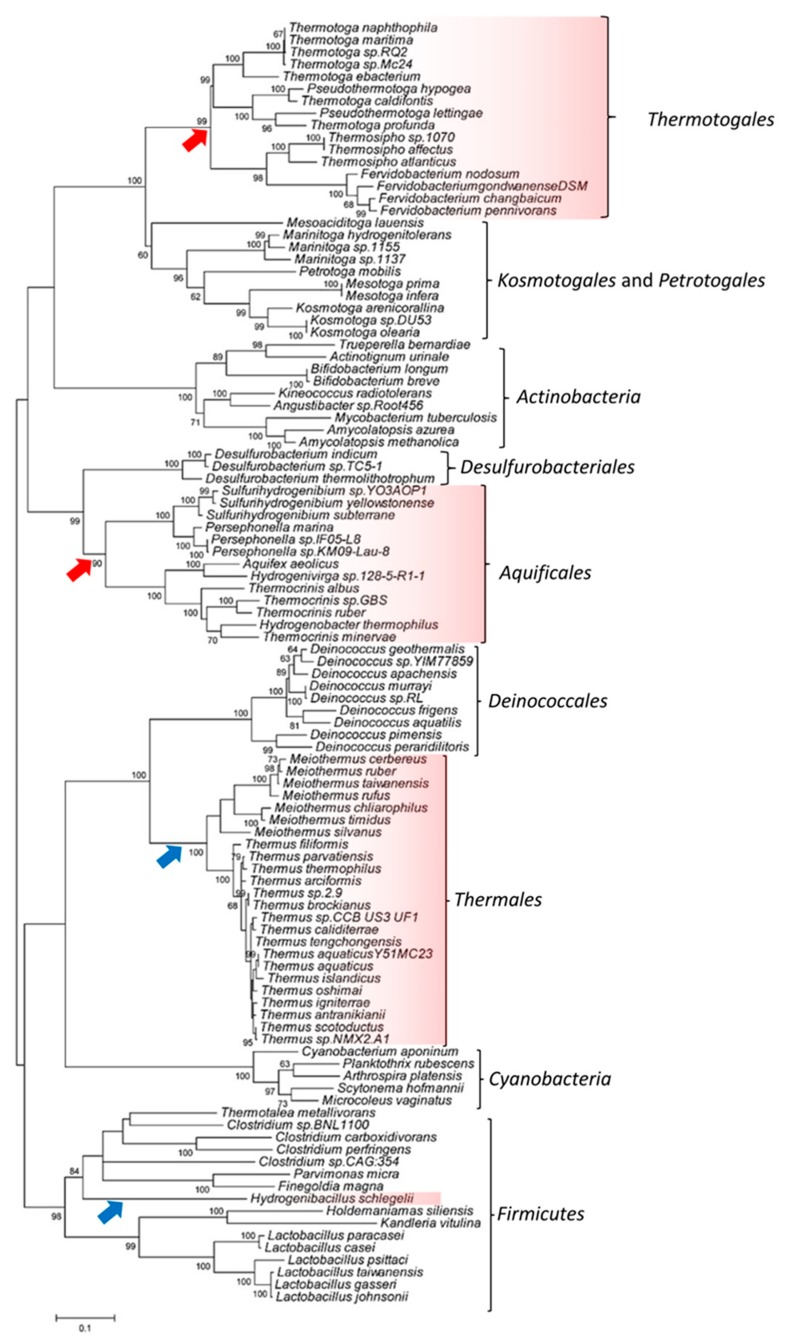
A maximum-likelihood phylogenetic tree based on SecA protein sequences from representative bacterial phyla. The groups of species containing the 50 aa conserved signature inserts (CSI) are marked by red arrows, whereas those containing the 76 aa CSI are denoted by blue arrows. The numbers on the nodes indicate bootstrap values for the observed groupings.

As both these large CSIs are present in only the thermophilic members from two different phyla of bacteria, it was of much interest to investigate how the shared presence of these genetic changes in two distinct groups/phyla of bacteria could be explained. Based on earlier work, horizontal gene transfers are indicated to occur frequently between members of the bacterial phyla that contain thermophilic–hyperthermophilic organisms [20,26,28,70]. Thus, we have examined whether the shared presence of these CSIs in these two cases is due to horizontal transfers of SecA gene between the two groups of organisms which contain either the 50 aa or the 76 aa inserts. To investigate this, we have constructed a maximum-likelihood phylogenetic tree based on SecA homologs from different relevant bacterial groups/phyla (Figure 3). In this tree, members from the phyla *Thermotogae* and *Aquificae* form distinct clades and the two orders of these bacteria viz. *Thermotogales* and *Aquificales*, which contained the 50 aa insert are separated from each other by other members of these phyla, which do not contain the 50 aa insert. Similarly, members of the order *Thermales* and the *Firmicutes* species *H. schlegelli*, both of which contained the 76 aa insert, also did not cluster together in the phylogenetic tree. Instead, members of the order *Thermales* branched with other members from the phylum *Deinococcus*–*Thermus*, whereas *H. schlegelli *branched within a cluster of other species from the phylum *Firmicutes*. If the shared presence of the 50 aa CSI and 76 aa CSI in the two indicated groups of organisms was due to horizontal gene transfers, then it was expected that the SecA genes from these CSI-containing organisms should have clustered together in the tree. However, as the observed branching pattern of the CSIs-containing organisms is contrary to this expectation, it strongly suggests that the shared presence of the large CSIs in either the order *Thermotogales* and *Aquificales,* or in *Thermales* and *H. schlegelli*, is not due to horizontal gene transfers. Instead the results obtained suggest that the genetic changes leading to these large inserts have occurred independently (i.e., convergent evolution) in these lineages due to their presumed selective advantage.

### 3.3. Computational Analysis of the CSIs in SecA Proteins

As the two large CSIs in the SecA proteins are found exclusively in the bacterial groups/orders that consist entirely of thermophilic and hyperthermophilic organisms, it strongly suggests that they play some role in the thermostability of the SecA protein. Hence, exploration of the functional characteristics of these CSI could provide some insights into the thermostability of the SecA protein. Based on earlier studies, thermostability of proteins is enhanced by an increase in the charged amino acids that facilitate increased ion-pair interactions and stabilize the protein in high entropy environment [11,13]. The results of our analyses (Figure S1) indicate that both the large CSIs in the SecA protein contains a higher proportion of charged amino acids such as Glu, Arg, and Lys, which are known to facilitate increased ion-pair interactions.

**Figure 4:**
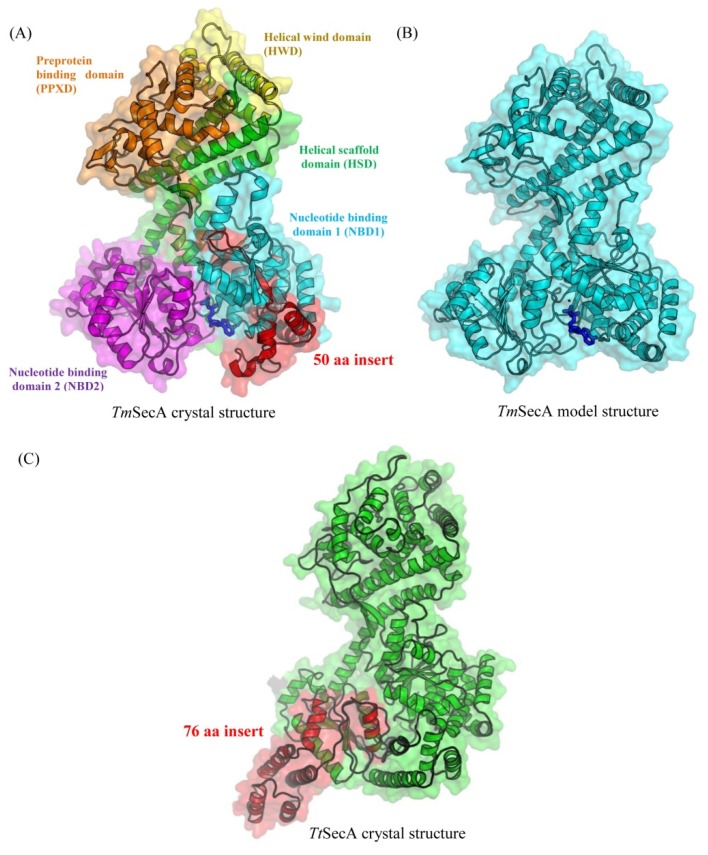
Cartoon and transparent surface representation of the 3D structure of (**A**) *Thermotoga maritima* SecA (PDB ID: 4YS0) shown as green and the various structural domains present in this protein are colored and labeled; (**B**) homology model of *T. maritima* SecA lacking the 50 aa CSI. The bound adenosine diphosphate (ADP) in the structure is shown as a blue stick; (**C**) Structure of SecA dimer from *Thermus thermophilus* (*Tt*SecA) (PDB ID: 2IPC). The 50 aa CSI and the 76 CSI are highlighted in red in these structures.

The crystal structures of SecA are available from both mesophilic bacteria (*Bacillus subtilis* and *Mycobacterium tuberculosis*), and from thermophilic bacteria (*Thermus thermophilus* (PDB ID: 2IPC) and *Thermotoga maritima *(PDB ID: 4YS0)) [36,37,71,72,73]. The catalytic core of SecA protein is comprised of five functionally essential domains, which are shown in Figure 4A in the available crystal structure of the *T. maritima* SecA (*Tm*SecA) [35]. The different domains of SecA protein are highlighted using different color shades: cyan as a nucleotide-binding domain (NBD1), magenta as nucleotide-binding domain 2 (NBD2), red as a pre-protein binding domain (PPXD), yellow as helical wind domain (HWD), and green as helical scaffold domain (HSD). We have also created a homology model of the *Tm*SecA protein lacking the 50 aa CSI, using the protocol described in the Methods section, for the comparative analyses of structural features and location of this conserved insert (Figure 4B). As can be seen, the 50 aa conserved insert in *Tm*SecA specific for *Thermotogales *and *Aquificales* is located in a surface-exposed loop region of the NBD1, which forms a part of the ATP-binding site in the protein [35,74]. The insert protrudes to form two additional β-strands at the periphery of the NBD1 domain with two short α-helices connected by a loop [37]. Although this surface-exposed CSI in the structure of *Tm*SecA is located in close proximity to the bound ADP molecule, it does not interact directly with bound ADP, nor it makes any contact with the SecY channel [37,75]. We have also mapped the location of the 76 aa CSI specific for the order *Thermales* and *H. schlegelii* using the available crystal structure of SecA from *Thermus thermophilus* SecA (*Tt*SecA) (PDB: 2IPC) [36]. As can be seen from Figure 4C, this large CSI is also located in a surface-exposed loop region of the *Tt*SecA at the periphery of NBD2. However, unlike the 50 CSI in *Thermotogales* and *Aquificales*, this CSI is not in close proximity to the ADP–ATP binding site on the protein. However, due to its location, this CSI, through its role in enabling intramolecular ionic interactions, could be playing a role in stabilizing dimer formation at higher temperatures.

### 3.4. Molecular Dynamics (MD) Simulation Studies of SecA Containing 50 aa CSI Specific for Thermotogales and Aquificales: Analysis of TmSecA Conformational Stability and Flexibility

In view of the location of the 50 aa CSI in the SecA of *Thermotogales *and *Aquificales* in close proximity to the ADP–ATP binding site, we have carried out MD simulation studies to gain some insights into the function of this CSI. In this regard, we have initially investigated the dynamic of the 50 aa CSI’s flexibility from the trajectories obtained from the MD simulation studies on *Tm*SecA structure with CSI (+CSI) and without CSI (−CSI) at two different temperature settings. The detailed protocol of the system setup for MD simulations is described in the Methods section. In total, four simulation runs were carried out using *Tm*SecA (+CSI) and *Tm*SecA (−CSI), each for 100 ns at two temperature settings of 303.15 K (30 °C) and 363.15 K (90 °C). A preliminary analysis was carried out to analyze the overall deviation of *Tm*SecA (+CSI) and *Tm*SecA (−CSI) relative to their native structures as a function of time using the trajectories along the 100 ns time scale at these temperatures. At 303.15 K, *Tm*SecA (+CSI) appear relatively more stable with an average RMSD of <0.20 nm (<2.0 Å) (±0.04 nm) relative to 0.39 nm (3.9 Å) (±0.08 nm) for *Tm*SecA (−CSI) (Figure S2A). Similarly, at 363.15 K, an average RMSD value of 0.34 nm (3.4 Å) (±0.5 nm) was observed for *Tm*SecA (+CSI) relative to an average value of 0.36 nm (3.6 Å) (±0.6 nm) for its insertion truncated homolog *Tm*SecA (−CSI) (Figure S2B). Although at the high temperature the differences in RMSD values are minimal, *Tm*SecA (−CSI) lacking the CSI showed a much higher degree of fluctuation, in comparison to the protein with the CSI, indicating that the *Tm*SecA (+CSI) was more stable at the higher temperature over the simulation period.

### 3.5. Identification of Conserved CSI-mediated Water Network in TmSecA

The crystal structure of *Tm*SecA with ADP-bound contains a number of bound water molecules near the ADP-binding site [35]. Of these water molecules, few forms an intermediate interaction between the adenine group of ADP to the backbone of the residues (Glu 185 and Val 186) from the 50 aa CSI in *Tm*SecA which is located near the ADP-binding site. This observation is of much interest, as several earlier studies indicate that water molecules make significant contribution towards the binding affinity of the ligand or mediating protein-ligand complexes by forming bridges between the protein and the ligand [76,77,78,79,80]. To investigate this, using the protocol described in the Methods section, computational analyses of the MD simulation trajectories were carried out to determine whether the 50 aa CSI in *Tm*SecA might be interacting with the ADP molecule by forming intermediate interactions with water molecules.

Initially, we analyzed the hydration of the ADP-binding site by calculating the presence of a number of water molecules within the 9 Å from ADP during the entire simulation of 100 ns at 303.15 K and 363.15 K (Figure S3). At 303.15 K, the increase in the number of water molecules increased in case of *Tm*SecA (+CSI) after 50 ns of the simulation, and overall it contained more water molecules (with an average of total 129 molecules) when compared with *Tm*SecA (−CSI) that contained an average total of 103 water molecules. However, at 363.15 K, the number of water molecules around the ADP-binding site for *Tm*SecA (+CSI) decreased after 50 ns of simulation time to an average total of 103 water molecules, whereas their numbers in *Tm*SecA (−CSI) slightly increases to an average total number of 112 water molecules.

**Figure 5:**
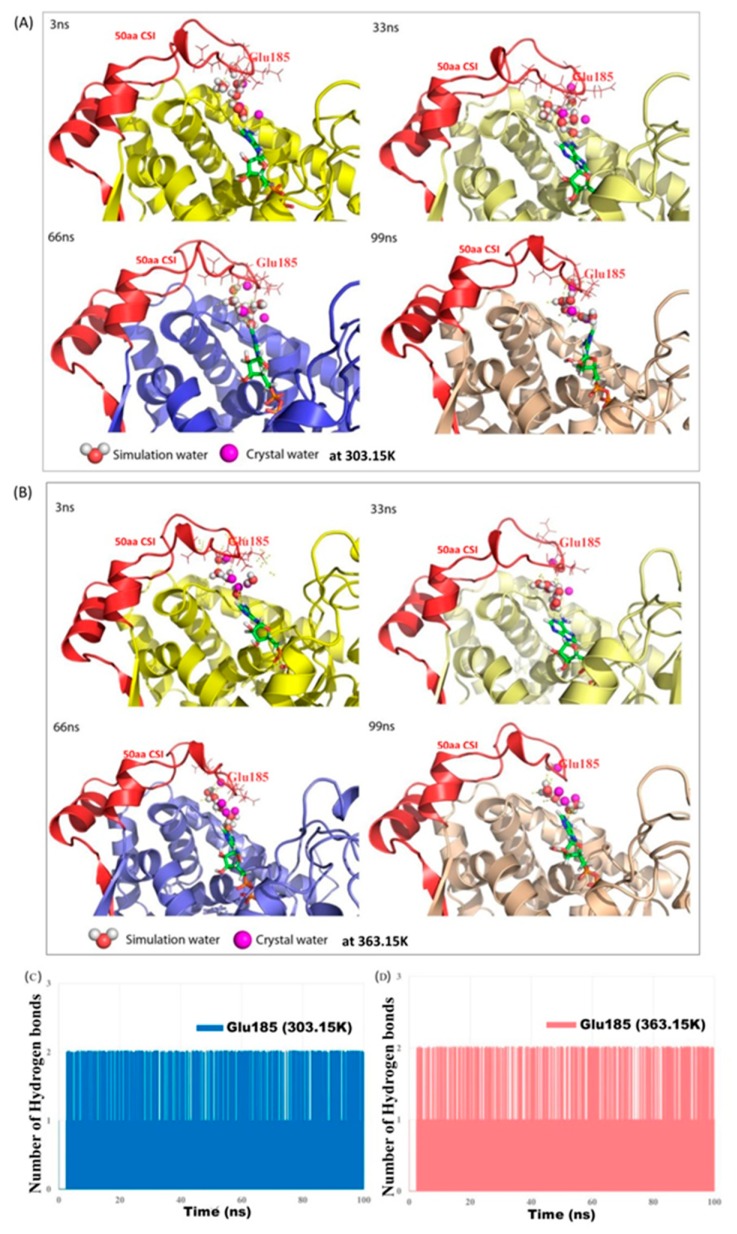
Snapshots from different time intervals extracted from the 100 ns molecular dynamic (MD) trajectories of *Tm*SecA (+CSI) showing the coordinates of water molecules from the simulation (red and white spheres) that constantly occupy the location near the backbone of residue Glu185 from the 50 aa CSI in *Tm*SecA at (**A**) 303.15 K, and (**B**) 363.15 K. Each of the extracted structures representing the different time scale is represented with different colors and the times are indicated in the snapshots. The magenta spheres represent the coordinates for crystallographic water molecules which are from the corresponding region in the crystal structure of *Tm*SecA and they are superimposed to each of the extracted structures to show the occupancy of the simulation water molecules of that region. Any hydrogen bonds formed between simulation water molecules and adenine group of ADP or residues from the insert are shown as yellow dash lines. Other randomly picked snapshots at different time intervals that show the occupancy and interaction of water molecules with residue (Glu185) from 50 aa CSI and ADP in *Tm*SecA are provided in Figure S4A,B. (**C**) Trajectories showing the time evolution of the number of hydrogen bond interactions formed between Glu185 residue backbone atom (O) from the 50 aa CSI and water molecules that are within 4 Å of Glu185 at 303.15 K (blue) and (**D**) 363.15 K (red), calculated over the 100 ns time period.

Further analyses of the MD trajectories identifies a network of water molecules that shows a high degree of conservancy and stable occupancy near the loop residues (amino acid range 183 to 188) from the 50 aa CSI and these water molecules formed interactions with the adenine group of ADP similar to that observed in the crystal structure of *Tm*SecA [35]. In a series of snapshots extracted at different time intervals from 100 ns MD trajectories of *Tm*SecA (+CSI) at 303.15 K (Figure 5A) and 363.15 K (Figure 5B), we show the coordinates of water molecules, which constantly occupy the location near the backbone of residues (amino acids 185–188) from this insert. For comparisons, observed positions of the water molecules in the *Tm*SecA crystal structure are superimposed in this figure and they are shown as magenta spheres. Any hydrogen bonds formed between simulation water molecules (red and white spheres), and adenine group of ADP or residues from the insert are shown as yellow dash lines. As can be seen, at both temperatures a network involving two to three water molecules are maintained throughout the simulation period. Although most of the water molecules are highly mobile, spending only a fraction of time in that position, interestingly the other water molecules that displace them occupy the same position and forms a network of water-mediated interactions that are highly similar to those observed in the crystal structure of *Tm*SecA protein [35].

The time evolution of the hydrogen bond interactions calculated between Glu185 and water molecules that are within the 4 Å of Glu185 over the course of 100 ns MD trajectory at 303.15 K and 363.15 K is shown in Figure 5C. It is of interest to note that the residue glutamic acid (Glu185) present in the loop region (residues 183–188) of this 50 aa insert is found to be conserved among SecA homologs from all members of the orders *Thermotogales *and *Aquificales*. As can be seen in Figure 5C, the backbone of Glu185 residue is involved in bridging the two hydrogen bond interactions with water molecules throughout the simulation at both temperatures.

## 4. Discussion

Thermophilic bacteria often exhibit many evolutionary adaptations that aids to retain the function of their proteins at very high temperatures [7,11]. These evolutionary adaptions such as ion-pair interactions, insertions and deletions, hydrogen bonds, and salt bridges ultimately result in increased intramolecular interactions and provide protein stability in the high entropy conditions [9,11,13,81,82]. In addition, a higher degree of close packing of water-accessible residues on the surface of the proteins and contact orders has been reported in thermophilic bacteria compared to their mesophilic homologs [7,16]. However, despite the significant progress that has been made toward understanding of the structural peculiarities of thermophilic proteins, no unique or single mechanism has been found responsible, instead, a set of factors or their various combinations has been suggested to contribute towards the thermostability of a protein [5,9,81,82,83,84,85].

In the current work, we describe the identification of two large and unique molecular sequence features in the form of CSIs that are uniquely present in the SecA homologs from different thermophilic and hyperthermophilic members from all three main phyla of bacteria (viz. *Thermotogae*, *Aquificae* and *Deinococcus*–*Thermus*), which harbor most such organisms [4,9,18,19,20,21]. Earlier studies on CSIs in different proteins show that these kind of rare genetic changes play important (or essential) roles in the functioning of the proteins within the CSI-containing organisms [66,86] and any significant changes in these genetic characteristics are incompatible with their cellular function/growth [86]. In view of the specificities of the identified CSIs in the SecA homologs for thermophilic–hyperthermophilic organisms, and the importance of such genetic changes, it is of much interest to understand what unique functions/roles these large CSIs play in the indicated groups/phyla of thermophilic bacteria.

Results presented here show that the two large CSIs in SecA protein are commonly shared by members from two different groups/phyla of organisms. While the 50 aa CSI in SecA is a uniquely shared characteristic of the members of the order *Thermotogales *and *Aquificales*, the 76 aa CSI is specific for the order *Thermales* and *H. schlegelii*, a species from the phylum *Firmicutes*. In both cases, the identified CSIs are present in two unrelated groups of bacteria in the same location in SecA protein. Further, these CSIs are of the same (or similar) lengths and exhibit a high degree of conservation in their amino acid sequences. As horizontal gene transfers among thermophilic organisms are indicated to occur frequently [20,26,28,70], the simplest explanations to account for the presence of these large genetic characteristics in members from two unrelated phyla of bacteria would be that the genetic changes leading to either the 50 aa or 76 aa CSIs initially occurred in one of the lineages of thermophilic bacteria and then the SecA genes containing these CSIs were laterally transferred to the other phyla of thermophilic bacteria containing very similar CSIs. However, the phylogenetic analyses of SecA protein sequences do not support the view that the shared presence of these CSIs in the unrelated groups of thermophiles is due to lateral gene transfers. To account for the observed results, it is likely that the genetic changes leading to these large CSIs have occurred independently in two unrelated groups of thermophiles due to selective (pressure) advantageous functions of these insertions in the growth/survival of indicated groups of thermophilic organisms at high temperature. However, given the large sizes of both these CSIs and the observed high degree of sequence conservation within their sequences, the possibility that these genetic changes have occurred independently in different lineages is unusual and unexpected. Hence, another possibility to account for these results is that instead of the horizontal transfer of the entire SecA genes, only the genetic exchanges or recombination of specific segments of SecA genes containing these CSIs have occurred between the indicated specific groups of thermophiles organisms [26]. As the rest of the SecA gene has evolved independently in these lineages, it will account for the distinct branching of organisms containing these CSIs in a phylogenetic tree based on SecA protein sequences.

It is of interest that within the phylum Aquificae, the 50 aa CSI is only shared by members of the order *Aquificales *but not by species from the order *Desulfurobacteriales*, which are also thermophilic. However, an important difference between members of the orders *Aquificales *and *Desulfurobacteriales* is that while the former order is comprised of species that are aerobic or microaerophilic and obtain energy from hydrogen or reduced sulfur compounds through molecular oxygen, the members of the latter order are strict anaerobes and they obtain energy by reduction of sulfate, nitrate, elemental sulfur, or other compounds by molecular hydrogen [20,29,87,88]. Additionally, the members of these two orders are known to possess different metabolic pathways as their environments have different metabolic requirements [20,65]. These observations suggest that the 50 aa CSI in the SecA protein may confer selective advantage (thermostability) only under aerobic conditions.

However, irrespective of the evolutionary mechanisms that underlie the shared presence of these CSIs, based on the specific presence of these large CSIs in SecA homologs from only the thermophilic-hyperthermophilic organisms, it is strongly anticipated that these CSIs should play an important role in the thermostability of this protein at high temperature. Results from our structural analyses show that both these CSIs are located on the surface-exposed loops in a functionally important domain (NBD1) of the SecA protein. Of the two CSIs in SecA, the 50 aa CSI, which is specific for the order *Thermotogales *and *Aquificales*, is located in a surface-exposed loop that lies in close proximity to the ADP/ATP-binding site in the protein. Our comparison of the crystal structure of *T. maritima *SecA containing the 50 aa CSI with its homology model lacking the 50 aa CSI has revealed that a number of water molecules form an intermediate interaction between the residues from this CSI and ADP molecule. Although the roles of conserved water molecules in mediating protein-ligand interactions have been increasingly recognized in recent years [76,78,79,80,89,90,91,92], our understanding of the significance of these conserved waters in SecA, or how other sequence features of the protein contribute towards their conservation, is limited. To investigate this aspect, molecular dynamics (MD) simulations were carried out to examine the structural dynamics of the 50 aa CSI in SecA protein and the water molecules found in its proximity in the crystal structure of *Tm*SecA at two different temperatures (303.15 K, and 363.15 K). The results from MD studies identify a network of highly stable water molecules that form an intermediate interaction between the residues such as Glu185 from the 50 aa CSI and adenine group of ADP at both temperatures. Earlier studies have indicated that the hydrogen bonding capability of water molecules with charged amino acids contributes towards the stability of hydrogen bonds formed between coenzymes/cofactors like ADP/ATP and proteins [14,93,94,95,96,97]. In view of these earlier studies, the high residence time of the water molecules inside the cavity formed by the 50 aa CSI, and their forming a conserved hydrogen bonding network with some conserved residues from the CSI during the course of simulation, strongly suggests that the 50 aa CSI likely plays a role in maintaining the constant stable network of water molecules which likely plays a role in stabilization of the ADP–ATP molecules in the active site of the protein at high temperature. Since ATP binding and hydrolysis is essential for the functioning of SecA protein, it is possible that the observed conserved hydrogen bonding network between the water molecules, residues from the CSI and ADP (ATP), helps in stabilizing the binding of ATP to the protein at high temperature.

However, the suggested role of the 50 aa CSI in stabilizing the binding of ADP/ATP to the protein at high temperature could be only one of the several factors by which this large CSI might be contributing towards the thermostability of the organisms for which they are specific. As noted earlier, these large CSI as well as the 76 aa CSI, specific to the member of the order *Thermales* and *H. schlegelli*, are present as surface-exposed loops in the structure of SecA protein. Based on earlier work, the surface loops in proteins are often involved in mediating novel protein-protein or protein-ligand interactions or in maintaining/stabilizing a specific oligomeric state of the proteins [45,46,47,86,98,99,100,101]. Earlier structural and functional studies also indicate that SecA can adopt a wide variety of oligomeric states [102,103,104,105] and it is possible that the presence of these CSI could stabilize certain oligomeric forms that are of relevance for the functioning of this protein in a thermophilic environment. In addition, most thermophilic and hyperthermophilic bacteria lacks SecB, a molecular chaperone protein which plays an important role in transferring pre-protein to SecA and SecYEG translocation system in most other bacteria [106,107,108]. Thus, it is likely that members of thermophilic and hyperthermophilic phyla utilize other chaperons or proteins for similar functions [107]. Although other novel proteins that may be required for the functioning of SecA protein in thermophilic organisms have not yet been characterized, it is possible that the presence of the unique surface-exposed loops formed by the CSIs in the SecA proteins from thermophilic organisms could serve as a platform for the unique binding of these proteins with the SecA. Overall, the results provided in this study highlights the unique sequence and structural features of SecA protein specific to the thermophilic and hyperthermophilic bacterial group. Our structural and computational analysis of these novel sequence features also provides some insights into the possible functions of one of these large CSIs in the context of the thermostability of the protein. However, further understanding of the functional significance of these large CSIs in the functioning of this protein in thermophilic organisms and conferring thermostability will only emerge from future detailed genetic and biochemical studies.

## Supplementary Materials

The following are available online at https://www.mdpi.com/2076-2607/8/1/59/s1. Figure S1: A Web logo representation of the sequence characteristics of the amino acids in the 50 aa and 76 aa CSIs. Figure S2: The root-mean-square-deviation (RMSD) values calculated relative to the starting structure for the *Thermotoga maritima *SecA (*Tm*SecA) with 50 insert (+CSI) and without 50 aa insert at 305.15 K and 363.15 K, over the 100 ns of molecular dynamics (MD) simulation trajectories. Figure S3: Trajectories for the occupancy of number of water molecules calculated by measuring the number of water molecules that are located within the 9 Å from ADP during the entire molecular dynamics (MD) simulation period of 100 ns. Figure S4A,B: Randomly picked snapshots at different time intervals extracted from the 100 ns MD trajectories of *Tm*SecA (+CSI) at 303.15 K and 363.15 K showing the occupancy and interaction of water molecules with residue (Glu185) from 50 aa CSI and ADP.
