# Supplementary material for: Novel Sequence Feature of SecA Translocase Protein Unique to the Thermophilic Bacteria: Bioinformatics Analyses to Investigate Their Potential Roles
Source: Microorganisms. 2019 Dec 29;8(1):59. doi: 10.3390/microorganisms8010059 (PMC7023208; doi:10.3390/microorganisms8010059)
Supplement: Supplementary file 1 [file microorganisms-08-00059-s001.pdf]

SUPPLEMENTARY MATERIALS

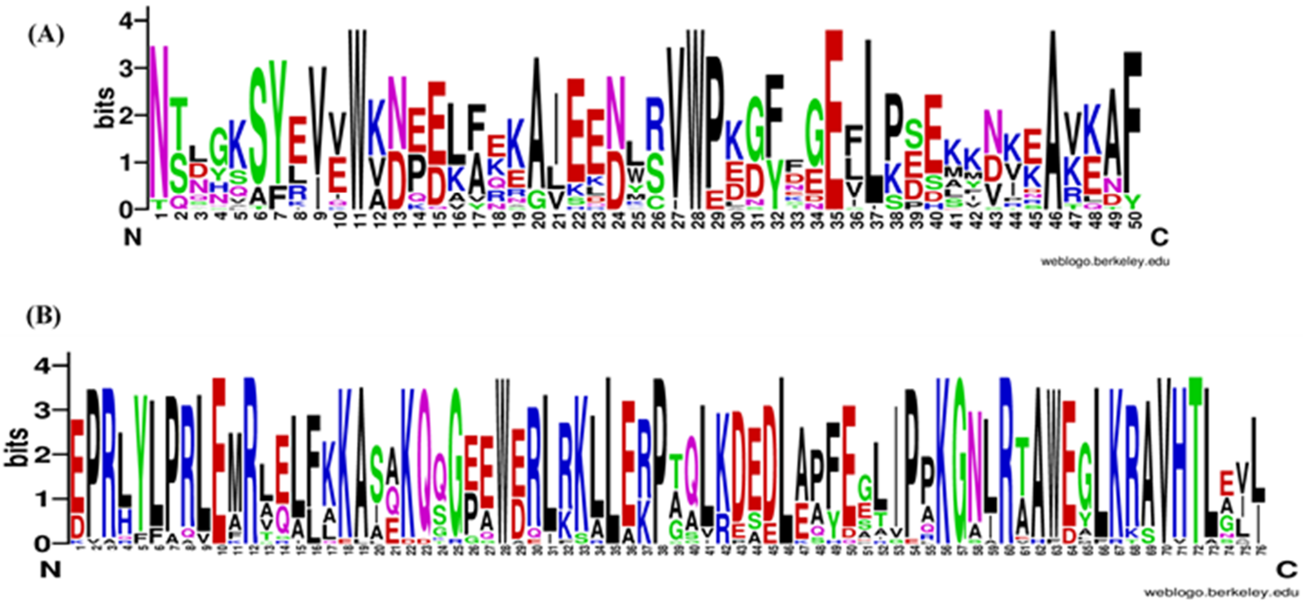

Figure S1. A Web logo representation of the sequence characteristics of amino acids in (A) 50 aa CSI and (B) 76 aa CSI. Web logos were created by using the CSIs entered into the Weblogo program (http://weblogo.berkeley.edu/) using default parameters. Amino acids are color coded according to their chemical properties as polar amino acids (G,S,T,Y,C,Q,N) are green, basic (K, R, H) are blue, acidic (D,E) are red and hydrophobic (A,V,L,I,P,W,F,M) are black.

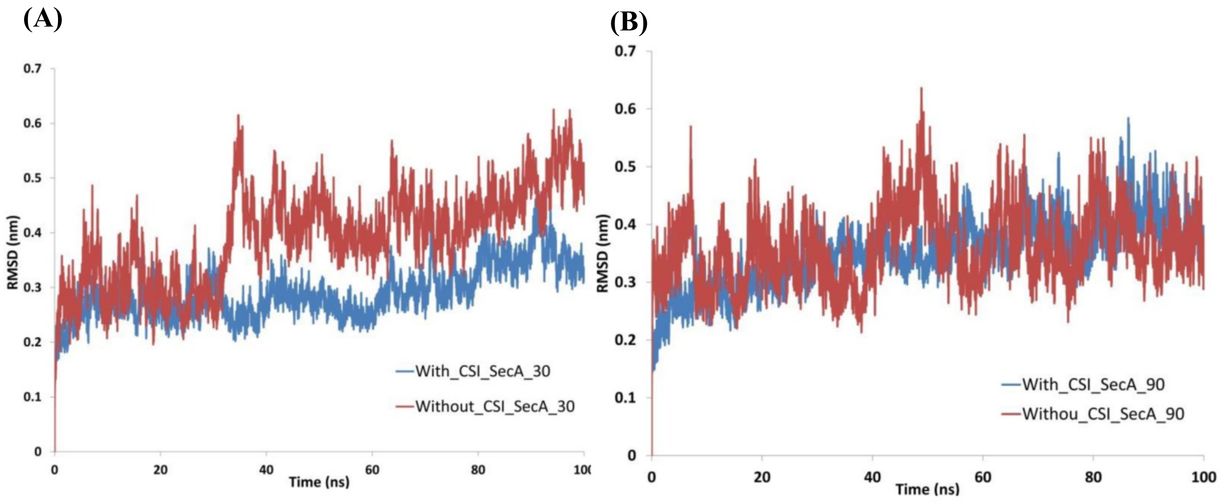

Figure S2. The root-mean-square-deviation (RMSD) values calculated relative to the starting structure for the *Thermotoga maritima* SecA (*Tm*SecA) with 50 insert (+CSI) and without 50 aa insert at 303.15K and 363.15K, over the 100 ns of molecular dynamics simulation trajectories.

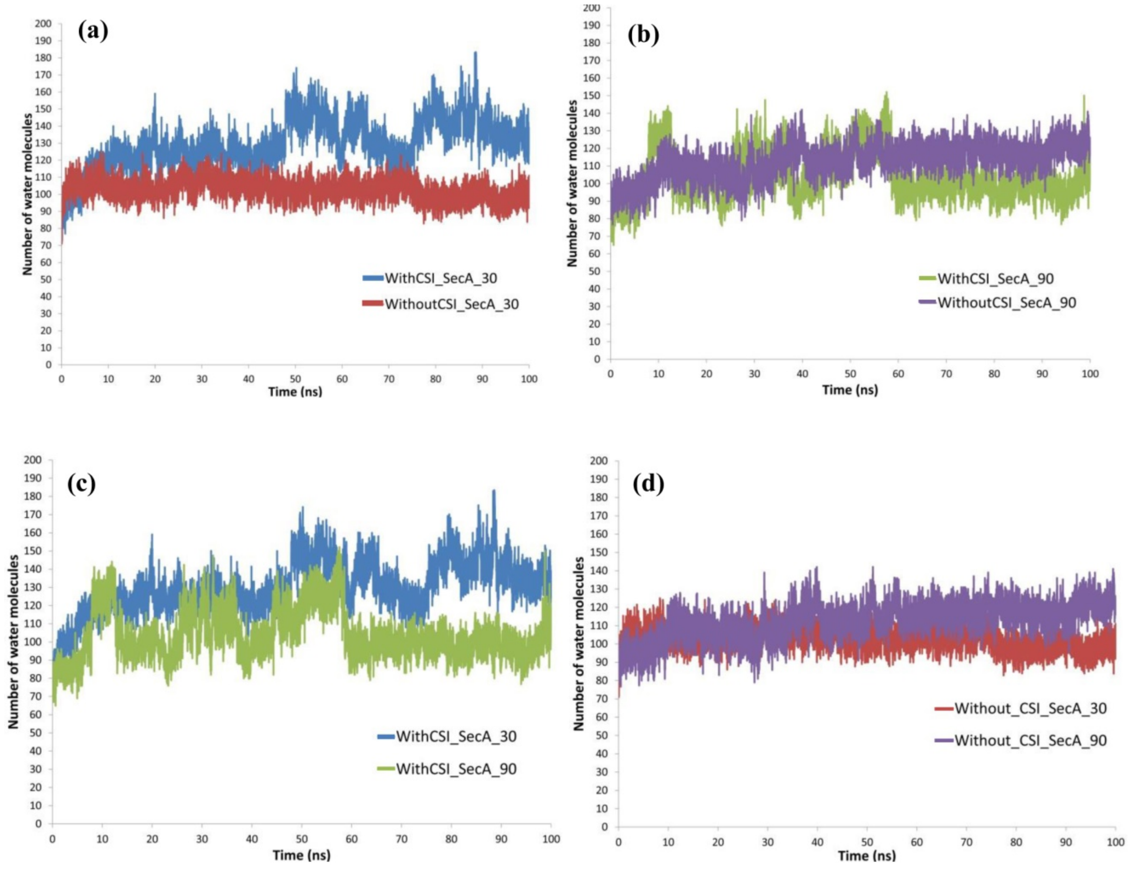

Figure S3. Trajectories for the occupancy of number of water molecules calculated by measuring the number of water molecules that are located with in the 9Å from ADP during the entire molecular dynamics (MD) simulation period of 100 ns. Occupancy of number of water molecules compared between the *Tm*SecA with (+CSI) and without (-CSI) 50 aa insertion at **(a)** 303.15K and **(b)** 363.15K. Comparisons of the number of water molecules calculated for **(c)** *Tm*SecA (+CSI) and **(d)** *Tm*SecA (-CSI) at two different temperatures as labelled in the plots.

A

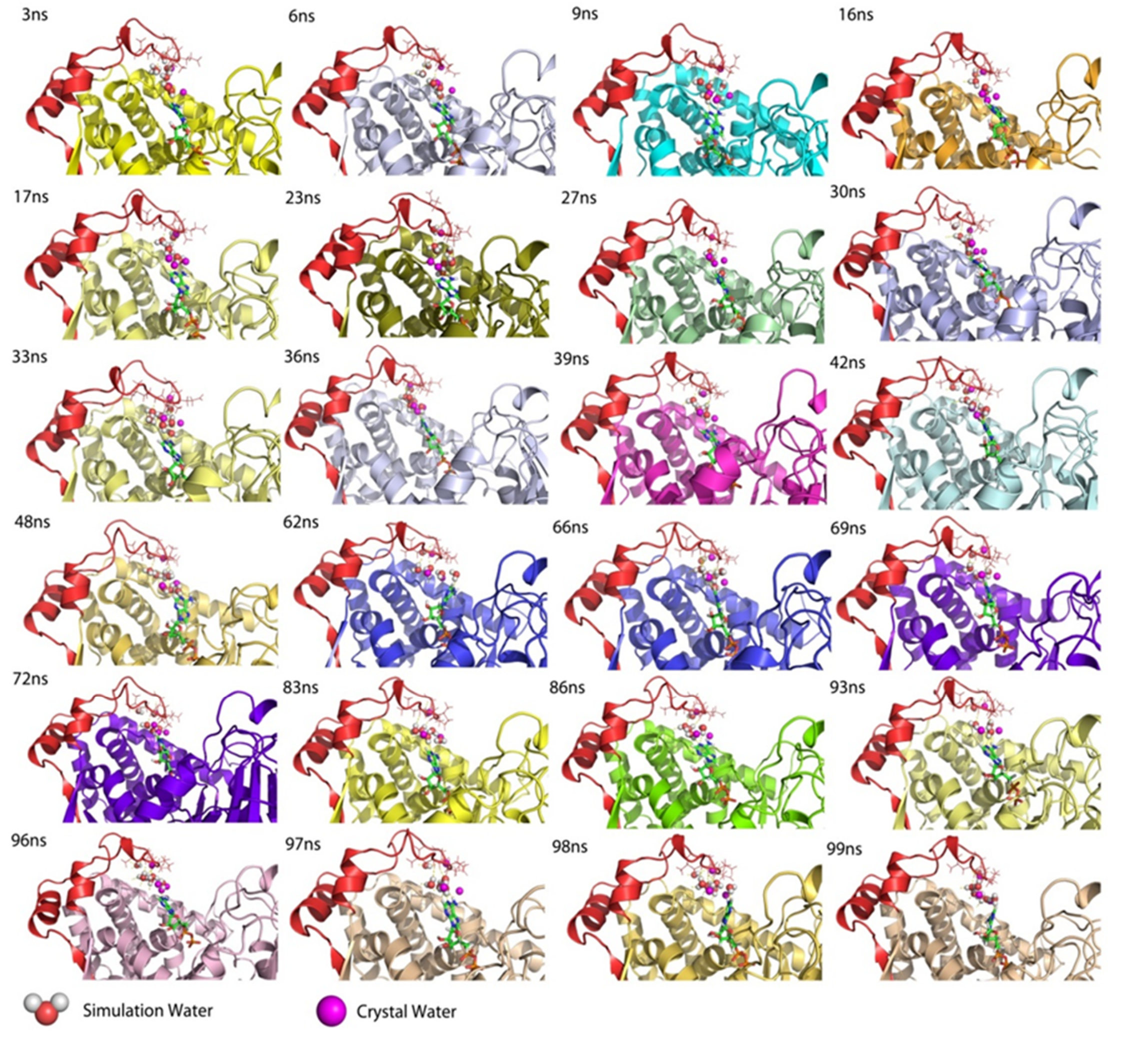

3ns
6ns
9ns
16ns
17ns
23ns
27ns
30ns
33ns
36ns
39ns
42ns
48ns
62ns
66ns
69ns
72ns
83ns
86ns
93ns
96ns
97ns
98ns
99ns
Simulation Water
Crystal Water

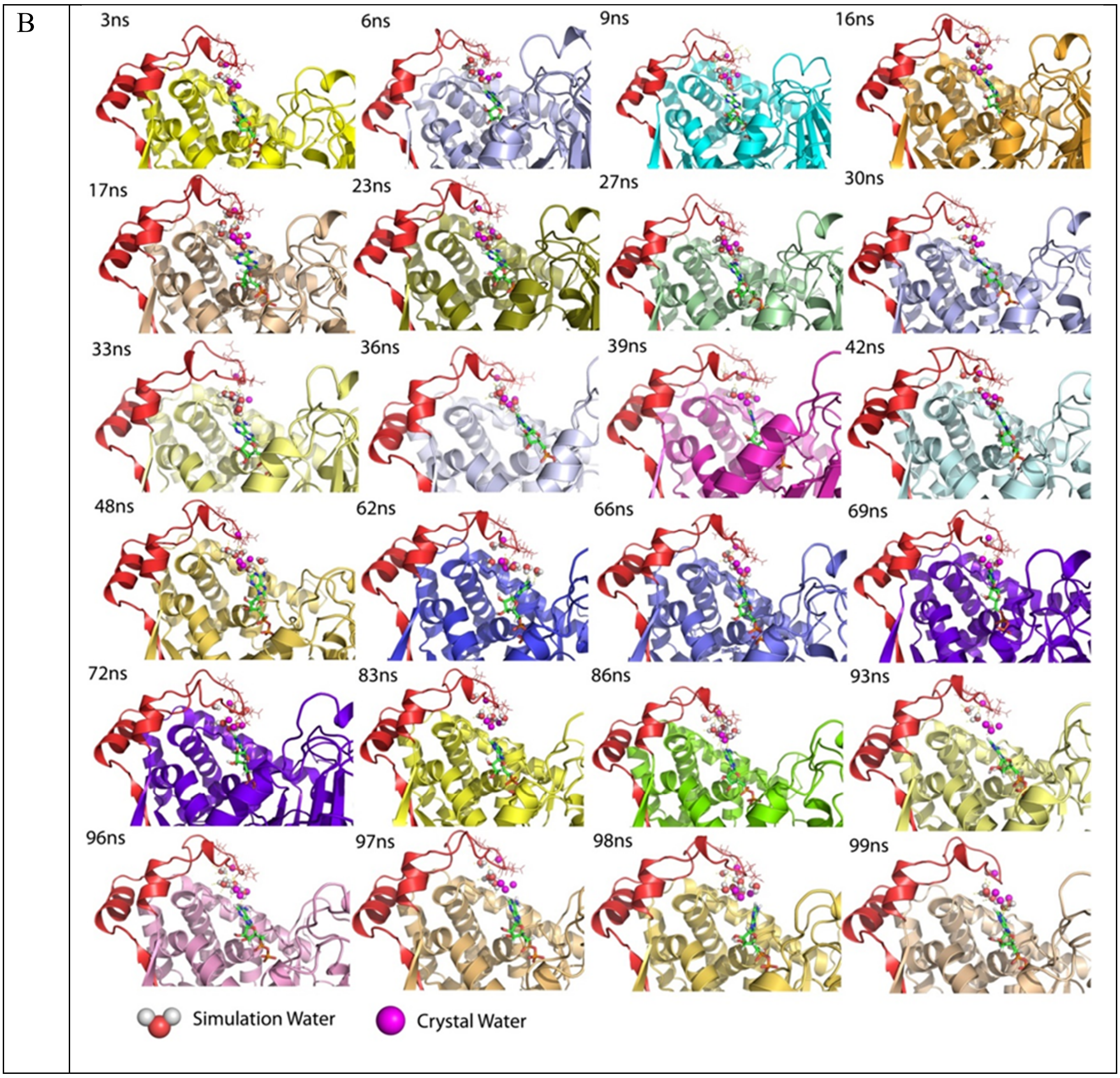

Figure S4. (A) Randomly picked snapshots at different time intervals extracted from the 100 ns MD trajectories of *Tm*SecA (+CSI) at 303.15K showing the occupancy and interaction of water molecules with residue (Glu185) from 50 aa CSI and ADP. **(B).** Randomly picked snapshots at different time intervals extracted from the 100 ns MD trajectories of *Tm*SecA (+CSI) at 363.15K showing the occupancy and interaction of water molecules with residue (Glu185) from 50 aa CSI and ADP.
